# Open data products-A framework for creating valuable analysis ready data

**DOI:** 10.1007/s10109-021-00363-5

**Published:** 2021-10-20

**Authors:** Dani Arribas-Bel, Mark Green, Francisco Rowe, Alex Singleton

**Affiliations:** Geographic Data Science Lab, Department of Geography and Planning, University of Liverpool, Roxby Building, 74, Bedford St S., Liverpool, L69 7ZT UK

**Keywords:** Geographic data science, Open data, Open source, C55, C63, C80

## Abstract

This paper develops the notion of “open data product”. We define an open data product as the open result of the processes through which a variety of data (open and not) are turned into accessible information through a service, infrastructure, analytics or a combination of all of them, where each step of development is designed to promote open principles. Open data products are born out of a (data) need and add value beyond simply publishing existing datasets. We argue that the process of adding value should adhere to the principles of open (geographic) data science, ensuring openness, transparency and reproducibility. We also contend that outreach, in the form of active communication and dissemination through dashboards, software and publication are key to engage end-users and ensure societal impact. Open data products have major benefits. First, they enable insights from highly sensitive, controlled and/or secure data which may not be accessible otherwise. Second, they can expand the use of commercial and administrative data for the public good leveraging on their high temporal frequency and geographic granularity. We also contend that there is a compelling need for open data products as we experience the current data revolution. New, emerging data sources are unprecedented in temporal frequency and geographical resolution, but they are large, unstructured, fragmented and often hard to access due to privacy and confidentiality concerns. By transforming raw (open or “closed”) data into ready to use open data products, new dimensions of human geographical processes can be captured and analysed, as we illustrate with existing examples. We conclude by arguing that several parallels exist between the role that open source software played in enabling research on spatial analysis in the 90 s and early 2000s, and the opportunities that open data products offer to unlock the potential of new forms of (geo-)data.

## Introduction

In the current era of digital transformation, data are a central pillar of the global economy and society. We have passed the point at which more data are being collected than can be physically stored (Lyman and Varian 2003; Gantz et al. 2007; Hilbert and López 2011).^1^ In addition to traditional forms of data, such as social surveys and censuses, major technological innovations have enabled an explosion in the generation, collection and use of new forms of data (Timmins et al. 2018). Networked sensors embedded in electronic devices, such as mobile phones, satellites, vehicles, smart energy meters, computers, GPS trackers and industrial machines can now sense, create and store data on locations, transactions, operations and people. Social media, web search engines and online shopping platforms have also spurred this data revolution by recording and storing users’ activity and personal information. Data are created as a by-product through interaction with these technological systems. While they are often not designed for research purposes, they can bring value for answering research questions (Timmins et al. 2018).

The world’s technological capacity to store, communicate and share information has significantly expanded. In 2018, companies worldwide were estimated to have generated and stored an excess of 33 zettabytes,^2^ seven exabytes of new data (Cisco 2018). Networked sensor technology in the financial services, manufacturing, healthcare and media and entertainment industries was estimated to account for 48 percent of global data generation globally in 2018 (Cisco 2018). In July 2019, 66 percent (over 5 billion people) of the world’s population were estimated to use mobile phones, 56 percent (over 4.3 billion) to be internet users, and 46 percent (over 3.4 billion) to comprise active social media users, whose penetration is growing at over 7 percent at year (Hootsuite and We Are Social 2019).

Despite the growing volume and speed of data collection and storage, only a small share are actually used. In 2019, a global study found that most organisations analysed less than half of the data they collected (Splunk, 2019). In 2018, a similar global survey estimated that 96% of all generated data in the engineering and construction industry goes unused (Snyder et al. 2018). In 2011, a small share of scientists from a survey of 1700 leading scientists reported to regularly use and analyse large data sets (Science Staff 2011). Only 12 percent reported data sets exceeding 100 gigabytes and use data sets exceeding 1 terabyte (Science Staff 2011).

The low utilisation rate of data may be reflective of barriers to access, as well as inability to process such vast quantities of information efficiently. Two key challenges involve privacy and confidentiality concerns, as well as the unstructured nature of data production and storage (Hanson et al. 2011; Manyika et al. 2015). Privacy and confidentiality concerns restrict access to data collected by companies and government agencies. The frequency, detail and geographical granularity of data being generated are unprecedented and therefore ensuring their privacy, confidentiality and integrity is critical. While legislation has been slow in responding to the changing landscape of digital data, it is now evolving in this direction. Major changes to ensure data protection and privacy were made to the EU General Data Protection Regulation (GDPR) which came into effect in 2018. Innovative institutional arrangements, such as data collaboratives (Verhulst, Young and Srinivasan 2017; Klievink et al. 2018) or services (e.g. Consumer Data Research Centre in the UK), have developed data sharing protocols and secure environments to facilitate access to commercial and administrative data for research purposes.

New forms of data are often highly unstructured and messy. They are produced in multiple formats, including videos, images and text; and, are stored in various organisational structures. Data are often not random samples of populations and are collected for specific administrative, business or operational purposes, and not necessarily for research (Hand 2018; Meng 2018; Timmins et al. 2018). In their original form, new forms of data are thus not readily usable limiting their applications. Significant data engineering is required, involving the use and design of specialised methods, software and expert knowledge, and linkage to other data sources (Hand 2018). To our knowledge, no formal analytical framework has been developed to chart the critical data engineering processes to develop purposely-built data products.

In this paper, we propose and develop the idea of Open Data Products (ODPs) as a framework to transform raw data into Analysis Ready Data (Giuliani et al.^2017^; Dwyer et al. 2018) and identify the key features that we contend of this framework. We define an ODP as the final data outcome resulting from adding value to raw, highly complex, unstructured and difficult-to-access data to address a well-defined problem, and making the generated data output openly available. Thus, three fundamental components characterise an ODP: its insightful utility, value added and open availability. We argue that an open data product has two major benefits. First, it enables developing insights from scattered, and/or highly sensitive, and/or controlled, and/or secure data which may be difficult to gather and use, or may not be accessible otherwise. Second, it expands the use of commercial and administrative data for the public good leveraging on their high temporal frequency and geographic granularity. We also contend that there is a compelling need for data open products as we experience the current data revolution. New, emerging data sources are unprecedented in temporal frequency and geographical resolution, but they are large, unstructured, fragmented and often expensive to assemble and possibly hard to access due to privacy and confidentiality concerns. By transforming raw (open or “closed”) data into valuable open data products, new dimensions of human geographical processes can be captured and analysed. Ultimately, ODPs may provide valuable guidance to develop appropriate policy interventions.

The paper is structured as follows: The next section defines and develops the idea of ODPs detailing the core elements to developing ODPs. We outline a framework that covers the initial conception of an ODP and moving through to developing and disseminating a product. Following, we discuss some of the challenges involved in the process of developing ODPs. The fourth section introduces some case studies of ODP exemplars which highlight the potential offered through our framework. Finally, we conclude the paper discussing the future potential of ODPs.

## Defining open data products

Defining Open Data Products (ODPs) is challenging since their remit is wide and incorporates several, diverse aspects. In some ways, they share several characteristics with traditional open data, as described in Kitchin (2014) or Janssen et al. (2012). To the extent ODPs result in open data, they also share most of their main benefits for society (Molloy 2011). It might be intuitive to assume that making Open Data 3 available that were previously not accessible would constitute an ODP. However, building ODPs is a broader project encapsulating frameworks for product development, such as data, delivery channels, transparent processes, etc. Indeed, ODPs adhere to standard principles of product development (e.g. Bhuiyan 2011) such as end user feedback or prioritising goals more than almost any other academic output.

In this context, we define ODPs as:

The open result of transparent processes through which a variety of data (open and not) are turned into accessible information through a service, infrastructure, analytics or a combination of all of them, where each step of development follows open principles.

We argue that the key difference between ODP over purely Open Data is the value added, which widens accessibility and use of data that would otherwise be expensive or inaccessible. Components of an ODP might include sophisticated data analysis to transform input data, digital infrastructure to host generated datasets, and dashboards, interactive web mapping sites or academic papers documenting the process. They almost always merge together data and algorithms but this is not necessarily a requisite.

While we adhere to general open principles, we recognise not all steps of the process can (or even need to) be fully open. We also argue a need for hybrid approaches that allow for closed data to be incorporated and opened up through the creation of ODPs (Singleton and Longley 2019). Such approaches are necessary for widening access to information derived from sensitive data. The resulting product should be released as open data; ideally too, the majority of the process that results in an ODP should be open, and although it might not be possible to release every component of an ODP, those related to infrastructure such as computer code, platforms and algorithms required to generate output data should be made available and transparent (Peng 2011; Singleton et al. 2016). Akin to the argument in open-source software, this is not only so that third parties re-run every step of the process again before using the data, but also to build a reproducible environment of trust that contributes to user adoption of the product’s outputs (Brunsdon and Comber 2020).

Although ODPs can take many forms and shapes, and hence differ greatly from each other, we think providing a few examples can be useful to land an abstract term in more practical settings. We will use two case studies that together embody differently but well both the ethos and the building blocks of ODPs: geodemographic classifications and data generated around the COVID19 pandemic. Below we introduce each, and we will return to different aspects in the next section.

Geodemographic classifications are created with the aim of describing the most salient characteristics of people and the areas where they live (Webber and Burrows 2018). There are various classifications spanning different countries and substantive uses across both the public and private sector (Singleton and Spielman 2014). Geodemographic classifications combine diverse sources of publicly and privately available data to generate insights about the behaviour of existing or prospective customers, service users and citizens. Technically, a geodemographic classification collates and combines disparate sources of data through a computational data reduction technique called cluster analysis that groups areas into a set of representative clusters describing salient patterns based on their similarity across a wide range of descriptive attributes.

Our second set of illustrations relate to the recent COVID-19 pandemic. The need to respond rapidly and efficiently to the spread of the virus, to save lives and sustain the economy, created intense demand for actionable data and information to feed into responsive decision making. Despite the global scope of the pandemic, many of the data generation, collection and processing systems originally in place were national at most, but in many cases regional or local. To bridge the gap between the available data and insight required, several researchers and organisations launched efforts to develop open data products. These included, for example, consolidated databases (e.g. Riffe et al. 2021) as well as ODPs derived from advanced analysis (e.g. Paez et al. 2020).

## The building blocks of open data products

In this section, we outline the key components of our proposed framework for developing ODPs. First, ODPs are born out of a need or problem that needs insight and will inform many design choices. Once the need is clearly delineated, the ODP process adds value to existing data in ways that help meet the original need. Adding value usually takes two forms: potentially complex transformations, fusion and abstraction of the data in what we call Open (Geographic) Data Science; and outreach activities to ensure the original need is addressed with the maximum impact. Throughout these explanations, we illustrate key points with the geodemographics and COVID-19 case studies introduced above.

### Identifying a problem in need of insight

Inception of an ODP begins with the identification of a concept or idea to address some problem that requires insight. Developing meaningful products often requires thinking less about ‘what’ a product might be, and more about ‘who’ might use it and what they would want to know. As such, identifying end users, understanding opportunities for satisfying their needs and mapping such opportunities to what is possible with the available data, skills and resources available can help to focus ODPs, and maximise their relevance. We would like to highlight this stage is usually followed in the research process (i.e. thinking about the “research question”), but that is not always the case in processes that result in open data. In fact, several open datasets are explicitly released as a side effect of the data existing for other purposes, and their release does not always have a clear end goal. While this has sometimes spurred several innovations (e.g. smartphone apps as a result of transit data made available as open data), we want to stress that ODPs are most useful when designed for a purpose and to further a goal.

This process can be independent, however if possible co-designing products can be an effective approach. Co-design (or co-production) is the involvement of external partners within the research process to help create user-led and user-focused products (Ostrom 1996). It is not clear what activities might be considered co-design (Filipe et al. 2017), however this process does not necessarily have to be onerous. Building trust through collaborations can help to ensure relevant and impactful products (Klievink et al. 2018). Data or knowledge exchange can facilitate partnerships, as well as opening up new ODPs that often would otherwise have not been made available. Developing partnerships (termed data collaboratives) is relevant here which are cross-sector initiatives for sharing or developing new data products that add value to the work undertaken by each actor in the collaboration (Klievink et al. 2018).

The principles of co-design are not limited to the identification of the product. It applies to each part of our framework, and understanding the end user needs is core to designing a successful product.

Perhaps the clearest example of the importance of a problem needing insight can be found in the recent pandemic. Understanding the uneven impact of the pandemic on society requires information about how different demographic groups from a wide variety of geographic contexts are affected. However, very few readily available datasets exist to understand the dynamics on the pandemic as it unfolds across different countries and different age groups. To fill this gap, Riffe et al. (2021) introduce a global demographic database of COVID-19 cases and deaths, COVerAGE-DB, enabling cross-country comparisons in the experiences of the pandemic.

### Adding value

The development of ODPs is not merely about making raw data available, as data driven innovation is more than opening up availability and use of data (Klievink et al. 2018). A key tenant of ODPs is to process, analyse and build on the original data, resulting in analysis ready data^4^ (see, for example, the collection introduced by Zhu 2019). This enhances the value of the information and opportunities for insight. The added value of ODPs can be achieved through numerous strategies, although these should ideally be linked to the first step of the framework to maximise their utility.

Development of new ODPs that extend the uses of existing data create value through producing new information. Data analysis can extract useful information or process data to create a new resource that demonstrates clear value added. Sources that cannot be made available in their raw form (often due to disclosure control or commercial sensitivity) can be made openly available through processing and manipulating into new ODPs with data owner permission.

Improving usability of data can help increase access, particularly where data acquisition is costly, hidden or publicly unavailable. It can be more salient when data are already available. But utilising or processing the data requires advanced quantitative skills to derive information, and bridging potential skills and knowledge gaps can open up existing data to a much wider audience (Klievink et al. 2018). This is pertinent for lay populations who, if ODPs are combined with interactive visualisations and resources, can engage with complex data in ways that might otherwise be unavailable to them. In such cases, value is added through focusing on the needs of the end users.

Matching or linking records can bring added value to existing databases or resources. Data linkage is the process of merging two or more independent resources or databases together based upon matching on a set of shared identifiers (Harron et al. 2017). Given the inherent costs of producing resources or collecting new data to investigate a research question, linking two or more existing sources together that could not answer the question by themselves, but possess all of the necessary information between them may provide a more efficient solution (Harron et al. 2017). Even where data linkage is not the priority, ODPs should be set up to allow future linkage to other potential resources.

By generating analysis ready data, ODPs bridge the gap between useful but inaccessible data and user needs. In doing so, they unlock potential research findings that derive from analysis that relies on them, and can feed into decision making that encourage more evidence-based policy making. Geodemographics and other composite indices are an excellent example of adding value to existing datasets. These approaches manage to leverage information from multiple data sources, deriving summary measures of the latent information (Green et al. 2018; Vickers and Rees 2007) while preserving the confidentiality of the original data as required.

### Open (geographic) data science

Various sources of new data forms are available in a “half-cooked” state (Spielman 2017). They are not available in a form that would be useful or accessible for interested stakeholders. For instance, data such as open transport data are available through convoluted processes (e.g. APIs) that non-technical audiences are not able to easily access. Others, such as satellite imagery or air quality data, can be downloaded easily but their size, complexity and unstructured nature preclude wider use. Yet, others, such as purchase records from retailers, exist but have restricted access. Given the accidental nature of many of these data sources (Arribas-Bel 2014), few undergo thorough quality assurance and assessments for bias, completeness and statistical representativeness. This is an important feature which differentiates new forms of data from traditional census and survey-based sources, for which there exist reliable infrastructure and frameworks for analysis, publication and dissemination.

The “unfinished” nature of new forms of data is a key feature of Data Science as a discipline. The explosion in the amount, variety and potential uses of new data has created the need for an interdisciplinary field that combines elements from areas such as statistics, computer science and information visualisation (Donoho 2017). Several new forms of data are inherently spatial, so there have been calls to establish closer links between these disciplines and Geography through GISc (Singleton and Arribas-Bel 2019), computational (Arribas-Bel and Reades 2018) and quantitative Geography (Arribas-Bel 2018).^5^This stage of the analysis has become increasingly sophisticated, increasingly with greater use of advanced algorithms and complex pipelines that transform data in useful ways. As an illustration, Stubbings et al. (2019) developed a green space index by combining street-level imagery, state-of-the-art deep learning techniques and hierarchical modelling. Dismissing this component of every data project as merely “data cleaning” involves several risks. It diminishes the credit awarded to a step that can crucially influence the final results, which compels researchers to relegate this key task to short and vague descriptions that obscure the steps undertaken, with clear implications for openness, transparency and reproducibility of their research (Brunsdon 2016).

We consider it vital that the (Geographic) Data Science process embedded in the generation of ODPs be as open and transparent as possible (Brunsdon and Comber 2020). Three main reasons underpin this requirement. First, as for open-source software (Raymond 1999), an open approach fosters collaboration, pooling of resources and avoids duplicating efforts. Second, an open approach involves an explicit recognition of the limitations of the datasets generated. Third, an open approach represents a clear message to users about the commitment to honesty and transparency by the ODP creator. This is an important element. The code, packages and platforms used to create an ODP will usually be accessed only by a small fraction of its users. However, the fact that they *can* be checked contributes to build user trust, and ultimately to amplify the use and impact of ODP by attracting a larger user base.

**Fig. 1:**
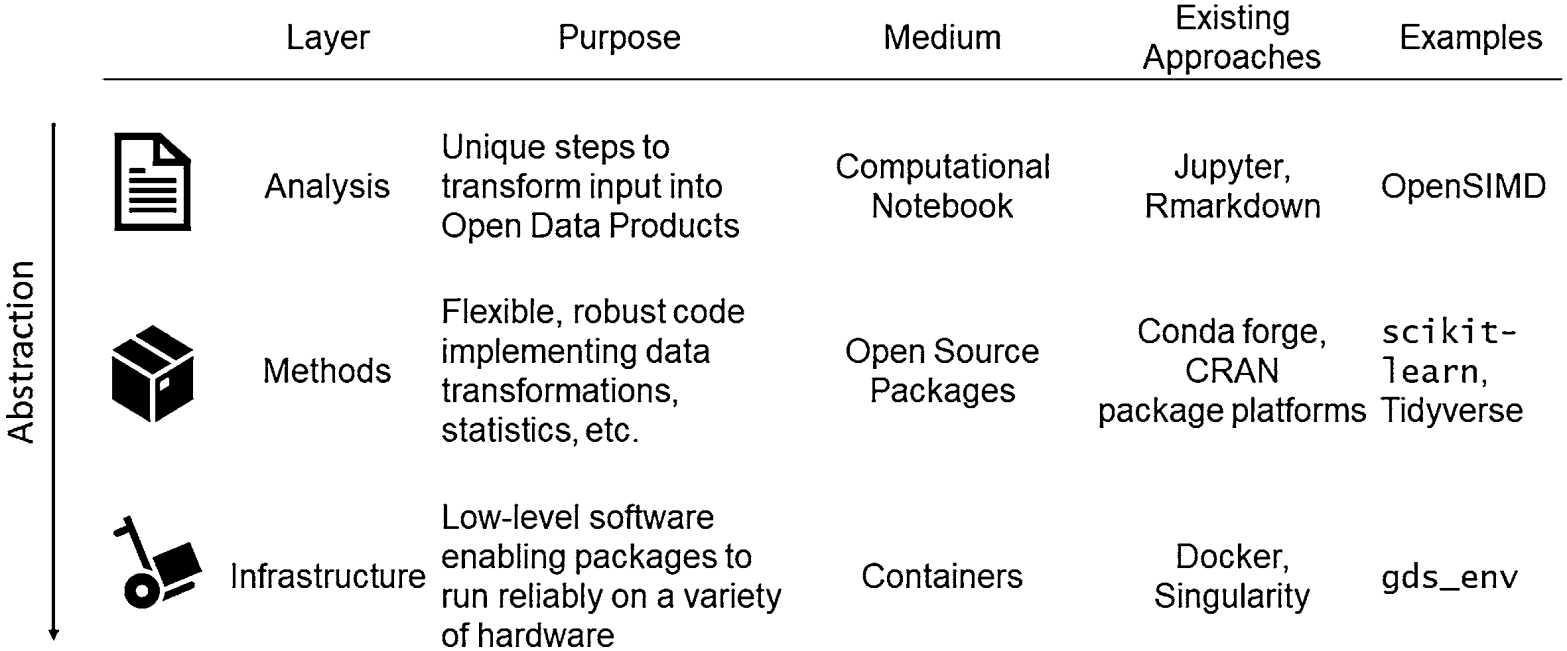
The geographic data science stack

The open approach that we recommend to maximise the impact of ODPs operates at three layers of the (Geographic) Data Science process: analysis, methods and infrastructure. Figure 1 shows an overview of what we term the Geographic Data Science stack. The top layer involves specification of the steps taken to transform the original input data into a final ODP, which we term ‘*analysis’*. In this context, the growing usage of computer code in research allows for the full documentation and evaluation of how products are developed (Brunsdon 2016). An open approach requires that the code generating the final dataset from the initial one(s) is available in both machine and human readable form. An increasingly popular format to meet this requirement within scientific communities is the computational notebook, such as Jupyter notebooks (Rule et al. 2019) or Rmarkdown notebooks (Casado-Díaz et al. 2017; Koster and Rowe 2019). In cases where commercial interest and copyright law prevents code sharing, so-called pseudo code with enough detail to reproduce the steps can be an acceptable compromise. Code released in the analysis stage should be specifically tailored to the development of the ODP. A good illustration of this approach is the Open SIMD project to expand on the Scottish Index of Multiple Deprivation (https://github.com/TheDataLabScotland/openSIMD).

The second layer involves *methods*. More generalisable code to implement a technique that could be applied in different contexts is relegated to this level. In this case, an open approach requires methods to be packaged as an open-source software library and released following standard software engineering practices (e.g. version control and continuous integration; Wolf, Oshan & Rey 2019). This division between analysis-specific code in notebooks and more modular code into packages avoids duplication of effort and increases the clarity with which the analysis is presented. Both R (CRAN) and Python (Conda-forge) are good examples of community approaches to support packages; similarly, projects such as scikit-learn (Pedregosa et al. 2011) or the Tidyverse federation of packages (Wickham et al. 2019) are good illustrations of open source packages.

The third layer comprises *infrastructure.* The growing complexity of modern software stacks and analysis pipelines requires open access to analysis and methods used, as well as the *infrastructure* on which the development of ODPs has been based be transparently detailed. In this context, ODPs can borrow from several advances in software development to make the data available. A prominent example is containerisation, the technology underpinning projects like Docker or Singularity, that allows to isolate the computational environment required to reproduce a set of commands. The gds_env project (Arribas-Bel 2019) provides an illustration for the case of GDS.

Full reproducibility may not always be possible or even desirable. For example, sensitive input data may not be amenable for sharing due to disclosure risks. We argue that as much of the process from start to finish should be made available, especially when there are few barriers against it. The purpose of an ODP is to design products that add value to existing data through opening up opportunities within data that are messy or unable to be openly shared.

A good example of the value of open geographic data science can be found in the geodemographics literature. Many of the original classifications were created by the private sector, where full disclosure of the underlying methods and data input is not always be possible given associated commercial sensitivity or intellectual property. Such an approach has drawn criticism as being “black box” (Singleton and Longley 2009). Arguably, this poses an acute issue for applications in the public sector, especially where life outcomes are at stake (Longley 2005). Responding to these concerns, there has been movement towards creating geodemographics that are more open to scrutiny. Under the umbrella of Open Geodemographics, several classifications that are fully reproducible have been created in countries such as the UK (Vickers and Rees 2007; Gale et al. 2016; Martin et al. 2018) and US (Spielman and Singleton 2015). In these instances, code and data are disseminated openly, and these academic outputs also have associated journal articles in the peer reviewed literature. Such an approach was made possible through all of the data integral to these classifications being disseminated with open licences and enabling reuse and redistribution.^6^More recent research also discusses alternative reproducible methods that might also be applicable when data are sourced with wider and more restrictive licensing arrangements where full reproducibility was not possible (Singleton and Longley 2019).

### Outreach

The mantra ‘build it and they will come’ should not be the outcome of ODP development. Successful dissemination, circulation and impact should not rely on chance. Outreach activities and resources are required to encourage end users to engage with a product. These activities should be designed to guide end users on the use of the ODP. A full review of various forms of outreach activities is beyond the scope of the paper, we focus on two main dissemination channels. It is important to recognise that several of these practices closely relate to and take inspiration from a variety of literatures, including those of participatory GIS (Dunn 2007) and citizen science (e.g. Haklay 2013).

A first key channel is user-focused events. These serve the purpose of refining and promoting a product. They can involve small, focused events such as workshops with stakeholders or lay community groups, and larger public promotion campaigns. Online presence and social media can play an important role in accessing wider coverage if supported with resources and materials. Project-specific social media accounts and online presence are increasingly more common. For example, the European Commission devotes an entire website to different aspects of their Global Human Settlement open data product.^7^Partnerships can also assist in the outreach process, especially when ODPs are designed to address a particular problem. For example, the “Access to Healthy Assets and Hazards” project (AHAH, Green et al. 2018) partnered with Public Health England (PHE) to make some of the data available through PHE’s Public Health Profiles resource. Co-designing an ODP requires engagement and co-development of project ideas with end users at every step so that the impact of ODP is maximised. Singleton and Longley (2019) co-developed a bespoke workplace classification in close collaboration with the Greater London Authority (GLA). The ODP is now available openly through the Consumer Data Research Centre’s data repository,^8^, and the GLA is using it for internal operations.

A second major channel involves the use of open-source platforms, software and resources. The integration of these assets is key to ensure interaction and engagement of end users with the ODPs, and a key principle is to facilitate end users with non-technical skills to interact with ODPs. Data stores comprise a useful example to make available ODPs and associated meta-data. Publishing all technical details, analytical code and documentation is important so that users can evaluate how ODPs were created and refine the project pipeline (see Paez et al. 2020, for an example of extensively documented data processes). Open-source platforms can help with this process, for example, CKAN for publishing open data, or GitHub for sharing code. Complementing these platforms should be the use of interactive resources that improve the accessibility and usability of ODPs. Examples of this approach include AHAH or the classification developed by Rowe et al. (2018) to analyse the trajectory of socio-economic inequality at the neighbourhood level in UK. These resources comprise an interactive web mapping tool that has been used by the general public and policy makers to point and click to their local areas and engage with the resource, as well as allow more technical users to download and analyse the information.

Journals have also emerged as a key mechanism for the explicit dissemination of ODPs. Innovative examples include Data in Brief,^9^ Scientific Data^10^ or REGION,^11^which publish papers explicitly focused on ODPs rather than focused on research from which a side product is an ODP. In doing so, they seek to promote the creation, sharing and reuse of scientific data. Papers are peer reviewed and published under an open license. This form of publication is useful as it provides essential context, describing how ODPs have been generated as well as assessing their limitations and identifying potential purposes for the reuse of generated ODPs (e.g. Rowe et al. 2017), all elements hard to cover on a traditional research paper. Journals, such as REGION, have also started publishing computational notebooks, and a key aim is their added value in communicating and disseminating ODPs (Koster and Rowe 2019). Notebooks offer interactivity with the potential to engage policy, discipline-specific or local knowledge experts with data analysis exploration (Rowe et al. 2020). This in turn can enable the identification of new relevant patterns or uses that may have not been reported or explicitly discussed in the original publication. These novel ways of publication provide an incentive for researchers to generate ODPs.

Outreach does not mark the end of developing ODPs. It is a continual and circular process that should incorporate constant evaluation and refinements to a product. Ideally, as data are updated, new relevant sources become available, and feedback from end users is gathered, they should be incorporated to refine ODPs. Outreach should therefore be designed to maximise this refinement process, facilitating feedback generation from relevant users.

Examples of outreach into stakeholders and users can be found in geodemographics. Spielman and Singleton (2015) and Patias et al. (2019) produced open classifications for the US and UK, respectively. Through further interaction, engagement and outreach, the Location intelligence company Carto^12^ has integrated them into their portfolio of data offerings. For the initial release of the US classification, only a description of the group level (ten clusters) was included, but Carto developed new labels for the 55 cluster type level, making these available within the public domain, alongside integration into their mapping platform,^13^used by industry and government. Thanks to this effort, the original classifications are openly accessible via their API and can be viewed within an interactive map improving their ease of access, engagement and dissemination.

ODP development and outreach has also been instrumental in supporting responses to the COVID-19 pandemic. For example, the Local Data Spaces project in the UK saw researchers working with Local Government practitioners to co-produce data insights using data held in secure and centralised researcher data environments (Leech et al. 2021). The aim was to help Local Authorities access these data directly or undertake research on their behalf, allowing them to gain data insights from data they did not have access to (including timely COVID-19 data deposited by the Office for National Statistics (ONS) not available elsewhere). Through continual repeated meetings with the team, researchers were able to co-design how Local Authorities wanted ODPs shared. Short computational notebooks were one solution, embedding descriptive data analyses as ‘conversation starters’ to show what data insights could be produced and help Local Authorities see the ‘art of possible’ (rather than sharing analysis ready data initially). For example, through sharing notebooks mapping asymptomatic COVID-19 test site accessibility in Liverpool, Liverpool City Council asked where to locate new sites and the team were able then focus on generating optimised locations to improve access (Green 2021). The added value of using notebooks meant that any analysis run for a Local Authority could be replicated for any other the local area resulting in all Local Authorities benefitting from insights during the co-production process.

## Challenges

Open Data are a good example of a Public Good, being both “non-rivalrous” and “non-excludable.” Open Data are, however, not free. There are direct costs associated with their collection, extraction, preparation and release; alongside indirect costs such as the loss of potential income that might be realised through alternative licensing models (Singleton et al. 2016; Johnson et al. 2017). Moreover, their consumption does not necessarily contribute to their production. For example, we might use OpenStreetMap data and services, but never commit any new geographic features or corrections to this open map system. Although some costs might be argued as being written off over time, others remain in perpetuity such as the cost of data hosting or download bandwidth. Issues of this nature which are associated with Open Data are generally enhanced when they are productised, given the additional human resource burden required in their creation, and the generation of necessary meta data or reporting associated with their release, such as extensive technical briefings, or the preparation of linked academic publications. As with Open Data, the “value” of an ODP is not realised directly (as it is free at the point of use), and to balance production costs, this would only likely to occur if these enveloped accounting of indirect benefits. For example, within some sectors where funding may be limited, an ODP might replace limited or no insight; potentially returning various economic or social benefits. Where funding is less constrained, ODPs may add value vis-a-vis commercial offerings if the insights generated are unique or complementary (Johnson et al. 2017). Capturing such value in both instances is however complex and lies somewhat outside the scope of this paper. However, given the costs of Open Data and those additional burdens of ODPs, there does need to be strategic planning and thought associated with creating ODPs. We would argue that some strategies that have been adopted by the Open Source Software community might be applicable within the context of an ODP. This might include the sponsorship of ODPs by organisations who are benefiting from their availability, or the integration of ODPs into commercial software as a service platform (e.g. API). More specifically, organisations developing ODPs, might also supply these within a ‘freemium’ model where enhanced versions of ODPs might be provided as commercial offerings.

The creation of ODPs share similarities to those ways in which open source software are produced. It has been argued that major contributions to many open source software packages are in fact mostly a result of contributions from a more limited set of developers (Krishnamurthy 2005). In a similar vein, many ODPs are created as a result of individuals or very focused teams. As with open software where there are a narrow set of contributors, this creates a challenge for how ODPs can be maintained and updated over time. Low diversity in teams developing Open Source Software (OSS) has also been suggested to hinder creativity and productivity (Giuri et al. 2010), which we would also argue is applicable to ODPs. Given these issues with OSS,one way in which they can be sustained is through code sharing platforms, such as Github or Bitbucket, where new developers can find out about software, make contributions or fork developments (Peng 2011). We argue that such platforms are equally useful for the sharing of code and data associated with the development of ODPs. However, they are not designed specifically for this purpose, and in essence, features are repurposed from the software developer community. The size of data that can be shared within such platforms is often limited, and where more extensive storage is required, this becomes an increasing cost burden. Although explicit data sharing platforms have emerged (e.g. figshare.com, zenodo.org, datadryad.org, dataverse.harvard.edu), these tend to focus on dissemination or archiving rather than development. Such platforms are useful for the promotion of ODPs, but are limited in functionality to support the process of remixing or update (Singleton et al. 2016). We would argue that there is space for new platforms with features that are better tailored to the needs of ODP development, and much like Github or Bitbucket might reward users through public profiles detailing their contributions to different ODPs.

The extent to which any community of ODP developers might be formalised and developed akin to those established within OSS will be challenging given the positioning of this emergent area (Harris et al. 2014; Arribas-Bel 2018; Arribas-Bel and Reades 2018; Singleton and Arribas-Bel 2019). Such issues are accentuated within our current university curricula. Within the Quantitative Social Sciences and Statistics, focus tends to favour theory and applications of statistical models. Although the processes of software development are considered within Computer Science, these focus on applications rather the use of code in development of ODPs. Moreover, the recent rapid growth of Data Science has so far emphasised visualisation and new modelling techniques from the cannon of machine learning and artificial intelligence. We argue that there is clearly a role for the better embedding of ODP development both within curricula bearing components of Data Science.

Finally, for those involved in the production of knowledge through research, historically there would be limited value ascribed to the considerable extra efforts required to package and document outputs from research as ODPs (Singleton et al. 2016). Within systems where impact is valued or measured, we argue that this might support engagement for the development of ODPs given their utility as a route to stakeholder engagement.

## Conclusion

This paper introduces the concept of Open Data Product as a construct that lowers barriers for a wider audience of stakeholders to access and benefit from the (geo-)data revolution. The value in framing the challenge of making sense of new forms of data through ODPs resides in its comprehensive approach. We focus neither exclusively on technical issues, such as the current big data discourse; nor on governance and outreach solely, such as more traditional open data notions. Instead, ODPs recognise that turning disparate, unstructured and often sensitive data sources into useful and accessible information for a wider audience of stakeholders requires a combination of computational, statistical and social efforts. In doing so, we contribute to the Open Data literature by providing a framework that expands the notion of how Open Data can be generated and what can constitute the basis to generate open datasets, as well as how to ensure its final usability and reliability.

Although not fully developed in this paper, we see a clear parallel between ODPs and the role that open-source software played in democratising access to cutting edge methods and computational power in the 90 s and 2000s. Three decades ago, a series of technological advances such as the advent of personal computing and rapid increase of computational power (e.g. Moore’s Law) provided fertile ground for experimentation in the domain of spatial analysis. Initially, however, this field of experimentation was hampered by a landscape dominated by proprietary software that was restrictive to access. Besides the obvious monetary cost, commercial software restricted access to methodological innovations as it used to be oriented to profitable market areas. In this context, OSS contributed significantly to unlock much of the potential of new computers and helped spur an era of new research that would have not been possible otherwise.^14^

We see data, rather than computation, as the defining feature of the present technological context. To make the most of new forms of data, we need more than “just” OSS; hence the proposal for ODPs in this paper. However, we would also like to stress the relevance and crucial role that OSS has to play in a world where “raw data” are so distant from an “analysis ready data”. As highlighted above, ODPs can only succeed through a transparent process that can build trust among end-users. Without the ability that currently only OSS provides to access cutting-edge techniques and do so in a transparent way, it is difficult to imagine successful ODPs.

Rather than definitive, our hope for this paper is to be provocative. The current data landscape is in transition and is very likely that several innovations are still in the not-so-distant horizon. Hence, the notion of ODP will necessarily be an evolving one that adapts to changing conditions to remain useful and valuable. At any rate, we envision the need for novel approaches and mindsets such as those described in this paper only to increase in the coming years. There is much that the spatial analysis community holds to contribute to exploit the data deluge that is rapidly changing every aspect of society. New ways to communicate and deliver our collective advances in data intelligence and expertise to maximise societal impact are needed. We hope the ideas presented in this paper partially shape the agenda and, more generally, contribute to a wider conversation about our role in shaping this new world in the making.

## Footnotes

1 Lyman and Varian (2003), for instance, estimated that 5 exabytes of new data generated through electronic channels, such as telephone, radio, television and the Internet were stored globally in 2002 but that more than three times that amount (i.e. 18 exabytes) were produced and not stored. Gantz et al. (2007) estimated that the amount of digital data created and replicated (255 exabytes) exceeded the storage capacity available (246 exabytes) in 2007. Hilbert and López (2011) estimated that the global general-purpose computing capacity -as a measure of the ability to generate and process data- grew at an annual rate of 58 percent, while global storage capacity grew at an annual rate of 23 percent between 1986 and 2007.

2 One zettabyte is equivalent to 10^21^ bytes. To visualise this, it would take one billion 1 TB external hard drives to store a zettabyte of data.

3 Open Data have numerous definitions but commonly refer to data that are released into the public domain without restrictive licenses that prevent their reuse or inclusion in derivative products. Such data are differentiated from free data (e.g. Twitter/Facebook API), that may be restricted in terms of access limits, but also importantly in the purposes to which the data can be applied or used.

4 The term “Analysis Ready Data” finds its origin in the remote sensing literature. We use it in this context because we believe the challenges and benefits of processing data before they are made available to end-users are extend well beyond satellite imagery.

5 We argue that, in this context, the term “Geographic Data Science” is more appropriate to capture the set of practices that we want to refer to. For more details on the motivation, reasoning and justification, in particular to how this term relates to more established ones such as GIScience or Geocomputation, we refer the reader to Arribas-Bel and Reades (2018) and Singleton and Arribas-Bel (2019).

6 For example, the 2011 ONS Output Area Classification has a formal page on the Office for National Statistics website here: https://www.ons.gov.uk/methodology/geography/geographicalproducts/areaclassifications/2011areaclassifications.

7 https://ghsl.jrc.ec.europa.eu/datasets.php.

8 http://data.cdrc.ac.uk/dataset/london-workplace-zone-classification.

9 https://www.journals.elsevier.com/data-in-brief/.

10 https://www.nature.com/sdata/.

11 https://openjournals.wu-wien.ac.at/ojs/index.php/region/.

12 https://carto.com.

13 The Carto blog describing the work can be found here: https://carto.com/blog/demographic-clusters-segmentation-data-observatory/.

14 For a practical illustration of this statement, the reader is advised to examine the number of published papers that actively cite open-source software projects such as GeoDa (Anselin, Syabri & Kho; 2006); R’s spdep (Bivand et al. 2011); or PySAL (Rey & Anselin 2010).
